# Global transmission dynamics of antibiotic resistance genes: Foodborne pathways as the critical link to humans

**DOI:** 10.1016/j.onehlt.2026.101531

**Published:** 2026-07-25

**Authors:** Emmanuel Stephen Odinga, Okugbe Ebiotubo Ohore, Sanji Zhou, Zhao Ziyu, Zhang Hongrui, Guojing Yang

**Affiliations:** Key Laboratory of Tropical Translational Medicine of Ministry of Education, School of Public Health, Hainan Academy of Medical Sciences, Hainan Medical University, Haikou, Hainan 571199, China

**Keywords:** AMR transmission dynamics, Resistome signatures, Food, Human, Environment

## Abstract

The global spread of antimicrobial resistance (AMR) poses a major challenge to food safety and public health. To evaluate foodborne transmission as a critical pathway, we analyzed 713,343 *Salmonella enterica* genomes from the NCBI Pathogen Detection database across 164 countries. A total of 363,409 ARGs conferring resistance to aminoglycosides, tetracyclines, and β-lactams were identified. Source-specific resistome signatures were evident, with primary sources (human feces, animal waste) exhibiting the highest ARG abundance and diversity, followed by secondary sources (food products) and tertiary sources (environmental matrices). Structural equation modeling (SEM) demonstrated that the food chain is a key transmission pathway for ARGs to humans, with strong positive associations from environment to food (β = 5.320) and food to humans (β = 11.334), while the direct environment-to-human pathway showed a significant negative effect (β = −34.809). Additionally, strong interclass correlations (*r* ≥ 0.96) among ARGs suggest co-selection and horizontal gene transfer as major drivers of resistance propagation. Our findings further reveal pronounced geographic and ecological variability in ARG prevalence, with the United States, United Kingdom, and China accounting for the highest ARG burdens. These findings highlight the central role of foodborne transmission in AMR dissemination and highlight the need for integrated surveillance and interventions targeting agricultural practices, food production, and environmental contamination. Overall, our work provides insights into the ARGs connectivity between environment, food and humans, and could help identify strategies to prevent dissemination of antibiotic resistance.

## Highlights

• Tetracycline resistance gene *tet(A)* was the most widespread ARGs across all isolation sources globally.
• Global AMR gene prevalence varied by region and by isolation source.
• The main transmission sources of ARGs from the environment to humans are via food.

## Graphical abstract

**Figure:**
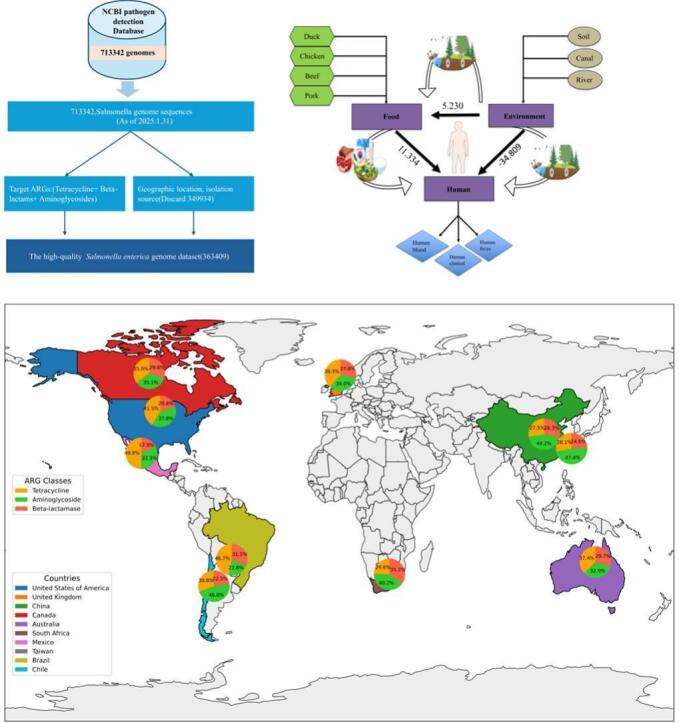

## 1 Introduction

Antimicrobial resistance (AMR) is a growing global health threat, undermining the effectiveness of antibiotics, complicating the treatment of infectious diseases and threatening food safety. One of the key drivers of AMR is the widespread dissemination of antibiotic resistance genes (ARGs) across different ecosystems, including environmental, agricultural, and clinical settings. These genes can be transferred horizontally among bacterial populations, promoting the emergence and spread of multidrug-resistant (MDR) pathogens with significant public health implications [44], [54], [62]. Addressing this challenge requires a comprehensive approach encompassing the monitoring of antibiotic resistance patterns, comprehension of resistance gene prevalence, and identification of potential sources and transmission avenues for resistant pathogens. Many studies have documented the presence of ARGs in isolated domains, such as in the environment, including soils, and aquatic ecosystems, or in food sources [41], [61] etc. However, the sole focus on quantitative or real-time polymerase chain reaction (qPCR)-based or metagenomics-based measurements of ARG abundances may result in the misidentification of many health-irrelevant ARGs. Neglecting the aggregate exposure pathways, which contribute to population-level acquisition of AMR, may lead to an underestimation of overall exposure. Therefore, it is imperative to translate these isolated resistome detection into real-life transmission pathway scenarios.

The environment plays a critical role in the AMR transmission cycle, agricultural practices, particularly the extensive use of antibiotics in livestock also contribute to the accumulation and amplification of ARGs in soil, water, and animal waste [[16], [34], [35], [63]]. These resistance determinants can enter the food chain through contamination during food production, processing, or distribution. Consequently, food products act as vehicles for the transfer of resistant bacteria to humans, representing a direct route of AMR exposure. *Salmonella enterica* is responsible for a substantial burden of foodborne illnesses globally, with increasing reports of multidrug-resistant strains associated with poultry and pork [22].These strains often harbor ARGs acquired from environmental or animal sources, underscoring the zoonotic and environmental dimensions of AMR spread [10].Notably, environmental contamination such as irrigation water tainted with animal waste can introduce resistant *Salmonella* into crops, creating a continuum of transmission that extends from soil and water to the human gut microbiota via the food chain [[17], [30], [36], [56]].

With the emergence and widespread occurrence of ARGs, *Salmonella* continue to pose serious public health concerns. AMR in *Salmonella* mainly varies with serotype, geographical location and source. For instance, AMR in *S. typhimurium* is relatively high compared to other serotypes such as *S. enteritidis*. However, in the U.S.A, a change in the dynamic of resistance in *S. enteritidis* was reported, with an upsurge in resistance to clinically important antimicrobials such as ceftriaxone and ampicillin [[14], [37], [38], [45]]. Since ARGs can be transmitted from the environment to humans via their pathogenic hosts, they can impact human health negatively [4], [24]. In most cases, ARGs move by horizontal gene transfer (HGT) from nonpathogens to pathogens, and this mechanism is a key driver in the evolution of pathogens resistant to antibiotics. It is important to note that only ARGs in pathogenic hosts can infect humans, thus posing a substantial threat to human health [[8], [9], [16], [28], [33]]. There have also been limited studies on how *Salmonella enterica* spreads resistance to humans globally. Therefore, it is important to understand the distribution and dissemination of ARGs in a global setting, especially their transmission from environmental matrices to humans [[20], [58], [59]]. This study hypothesize that host-specific factors determine resistome structure and profile, driven by differential resistance preferences and fitness costs. This host-associated resistome signature subsequently shapes the transmission pathways of ARGs from environmental reservoirs to food products.

Despite extensive reports of ARGs in different environments, a major knowledge gap remains: to what extent is foodborne transmission the central link between environmental resistomes and human health? Most studies focus on ARG abundances, but these do not directly translate into human exposure risks. Addressing this requires an integrated global analysis that considers ARG prevalence across sources and quantifies transmission pathways. Here, we analyzed >700,000 *Salmonella enterica* genomes to test the hypothesis that foodborne pathways represent the dominant route of ARG transfer from environmental and animal reservoirs to humans. By integrating source-specific resistome signatures with structural equation modeling, we provide new insights into how food systems mediate the global dissemination of AMR.

## 2 Materials and methods

### 2.1 Data acquisition and processing

In this study, *Salmonella enterica* isolate data were retrieved on 31st January 2025, from the NCBI Pathogen Detection (NCBI-PD) database (https://www.ncbi.nlm.nih.gov/projects/pathogens), which consolidates genomic sequences of bacterial and fungal pathogens from multiple active surveillance programs. These programs encompass diverse sources, including food products, environmental samples (e.g., water, production facilities), and clinical specimens. As part of the National Database of Antibiotic-Resistant Organisms (NDARO), the NCBI employs the AMRFinderPlus tool to systematically screen these genomic datasets for antimicrobial resistance (AMR) genes, stress response elements, and virulence factors. This integrated platform facilitates high-resolution tracking of resistance gene dissemination and enables comprehensive analyses of the interplay between antimicrobial resistance, stress adaptation, and virulence in pathogenic microorganisms.

A total of 713,343 *Salmonella enterica* isolates were retrieved and analyzed to investigate the distribution of antibiotic resistance genes (ARGs). Metadata for each isolate, including location, AMR genotypes, and isolation source, was obtained. Samples with missing metadata were excluded from the analysis. Finally, twenty-two ARGs representing three key antibiotic classes tetracyclines, aminoglycosides, and beta-lactams were selected for focused analysis based on their clinical and epidemiological relevance. Resistome sources exhibiting a cumulative frequency of occurrence greater than 50% were prioritized for downstream investigation and systematically categorized into three hierarchical groups: primary, secondary, and tertiary sources. Primary sources encompassed samples directly linked to living hosts, including human-related materials (e.g., urine, stool, clinical specimens) and animal-related materials (e.g., live animals, animal excretions). Secondary sources included consumables derived from animals, such as processed meat products (e.g., beef, poultry, pork), reflecting a critical interface between human and animal resistomes.Tertiary sources comprised environmental reservoirs, including agricultural soil, wastewater, and swabs from agricultural production environments (Table S1, S2 &S3). This classification enabled a structured analysis of ARG dissemination pathways across human, food, and environmental domains, providing insight into the potential reservoirs and transmission dynamics of antimicrobial resistance. Based on the aforementioned criteria, a total of 100 sources were obtained and merged into 14 distinct sources, to avoid repetition and redundancies.

### 2.2 Statistical analysis

Resistome source metadata were initially curated and categorized using Microsoft Excel (2010, Microsoft Corp., USA). All downstream statistical analyses and visualizations were performed using R software (version 4.4.3) and Python, employing the *pheatmap* package for heatmap construction and multivariate analyses[29]. Principal component analysis (PCA) and non-metric multidimensional scaling (NMDS) were applied to explore similarities and differences in ARG profiles across isolation sources.

To investigate transmission relationships among environmental, food, and human-associated *Salmonella enterica* resistomes, we employed structural equation modeling (SEM) using AMOS Graphics (version 24, SPSS, IBM, USA). SEM was selected because it allows simultaneous evaluation of multiple hypothesized pathways and indirect effects among latent variables representing ARG burdens in different compartments.

The model assessed three primary pathways: environment-to-food, food-to-human, and environment-to-human, with ARG abundance serving as the observed variable for each compartment. Model parameters were estimated using maximum likelihood estimation. Model performance was evaluated using the normed chi-square statistic (CMIN/DF) and the Comparative Fit Index (CFI). Values of CMIN/DF 0.00 and CFI ≥ 1.000 were considered indicative of acceptable model fit. The SEM was interpreted as a statistical representation of associations among *Salmonella enterica* resistomes across compartments rather than as direct evidence of causality.

## 3 Results

### 3.1 Global distribution patterns of the pathogenic bacteria and prominent ARGs showed source-specific resistome signatures

**Fig. 1:**
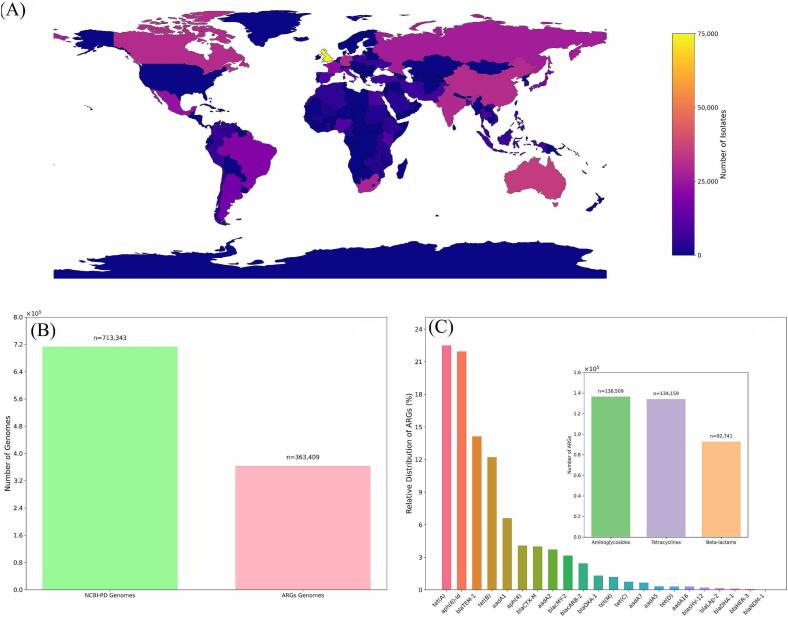
Global distribution and characterization of antibiotic resistance genes (ARGs) in genomic datasets. (a) Global prevalence of *Salmonella enterica* isolates, per country (ranging from 0 to over 75,000). (b)Total number of pathogenic genomes retrieved from the NCBI Pathogen Detection (NCBI-PD) database compared to the number of genomes containing identifiable ARGs. Out of 713,343 genomes, 363,409 were found to harbor target ARGs. (c) Relative distribution (%) of the most prevalent ARGs across all ARG-containing genomes. Total number of ARGs grouped by antibiotic class. Aminoglycoside resistance genes were the most abundant (136,509), followed by tetracycline (134,159) and beta-lactam resistance genes (92,741).

Our study utilized a dataset of 713,343 isolates to illustrate the global distribution pattern of *Salmonella enterica* and the spread of resistance worldwide. The isolates were obtained from diverse sources, including human, environmental, clinical, food, soil, river, and stool samples. The global distribution of *Salmonella enterica* isolates depicted significant variation across different regions, with countries such as the United States, United Kingdom, Germany and China reporting high numbers of isolates, with counts exceeding 50,000 or 75,000 while Brazil, India, and Australia showing moderate counts of isolates (25,000–50,000) (Fig. 1). Regions such as Africa, parts of Asia, and Oceania reported limited data which reflects either underreporting or insufficient surveillance, which raises concerns about the robustness of the data from these regions.

Among the selected resistance genes of interest, aminoglycoside (amg) genes (*n* = 136,509 ARGs) were the most predominant, followed by tetracycline (tet) (*n* = 134,159) and β-lactam (bla) (*n* = 92,741) resistance genes. The top five ARGs across all resistance gene classes included *tet(A)* (tet), *aph(6)-Id* (amg), *blaTEM-1* (bla), *tet(B)* (tet), and *aadA1* (amg), accounting for 23% (*n* = 81,749), 22% (*n* = 79,714), 14% (*n* = 50,877), 12% (*n* = 44,350), and 7% (*n* = 25,438) of all isolates, respectively. Within the tetracycline class, the overall distribution of resistance genes was as follows: *tet(A)* (23%, n = 81,749), *tet(B)* (12%, n = 44,350), *tet(C)* (0.74%, *n* = 2689), *tet(D)* (0.3%, *n* = 1080), and *tet(M)* (1.2%, *n* = 4291). Aminoglycoside resistance genes were also prevalent, with *aph(6)-Id at* 22% (n = 79,714), *aadA1* at 7% (*n* = 23,971), *aadA2* at 3.7% (*n* = 13,496), *aph(4)-Ia* at 4% (*n* = 14,777), *aadA5* at 0.3% (*n* = 1157), *aadA7* at 0.7% (*n* = 2411), and *aadA16* at 0.27% (*n* = 983). β-lactam resistance genes were also widely distributed, with *blaTEM-1* at 14% (*n* = 51,331), *blaCTX-M* at 4% (n = 14,484), *blaCMY-2* at 3.1% (n = 11,397), *blaCARB-2* at 2.4% (*n* = 8846), *blaOXA-1* at 1.3% (*n* = 4727), *blaSHV-12* at 0.2% (*n* = 717), *blaLAP-2* at 0.1% (*n* = 497), *blaDHA-1* at 0.1% (*n* = 401), *blaHER-3* at 0.08% (*n* = 289), and the carbapenemase *blaNDM-1,* although rare, was detected in 0.01% (*n* = 52) of isolates.

Collectively, these findings highlight the extensive and diverse resistome present within *S. enterica* populations. The widespread presence of high-priority ARGs, including those conferring resistance to critical antibiotics such as β-lactams and aminoglycosides, highlights the ongoing risk of resistance propagation and reinforces the need for continuous genomic surveillance and global mitigation efforts.

**Fig. 2:**
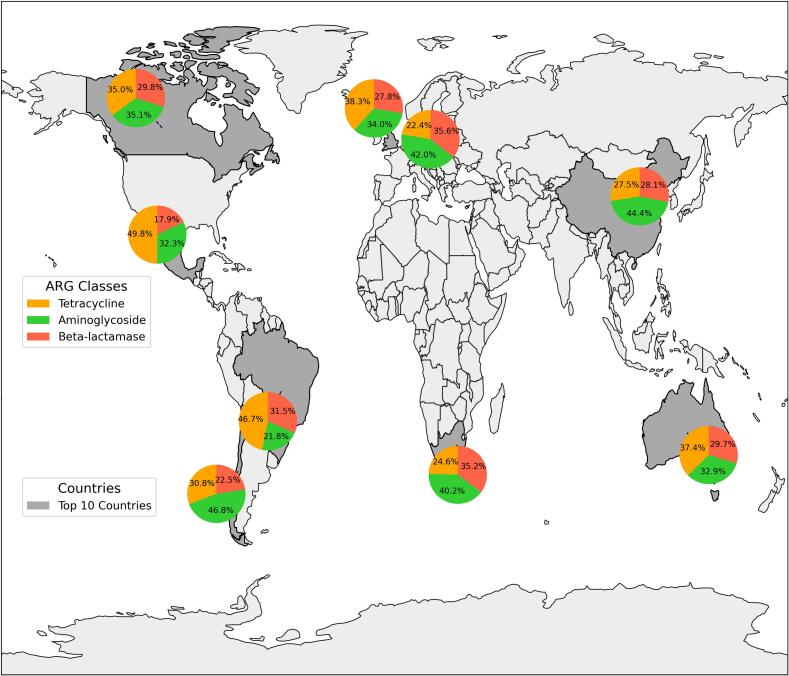
Prevalence of antibiotic resistance gene (ARG) classes across different countries*.* The pie charts represent the total percentage of each ARG class per country detected in samples from ten countries: United States, United Kingdom, Germany,China, Canada, Australia, South Africa, Mexico,Brazil, and Chile, highlighting variations in ARG distribution patterns, suggesting regional differences in antibiotic usage and resistance pressures.

To elucidate global distribution patterns of ARGs, we identified a total of 363,409 resistance genes from tetracyclines, aminoglycosides, and β-lactams, and investigated their prevalence across different countries. These ARGs were detected in isolates from 164 countries (Fig. S1). The top ten countries with the highest detection frequencies were the United States, United Kingdom, China, Canada, Australia, South Africa, Mexico, Brazil, and Chile (Fig. 2).

The United States had a total ARG count of 176,757, with the distribution of ARG classes dominated by tetracyclines (41.5%), followed by aminoglycosides (37.8%) and β-lactamases (20.7%). The United Kingdom had a total ARG count of 47,533, with aminoglycoside resistance genes being the most prevalent (38.3%), followed by tetracyclines (34.0%) and β-lactamases (27.7%). In contrast, China (39,822 ARGs) exhibited a higher proportion of tetracycline resistance genes (44.2%), with β-lactamases (28.3%) and aminoglycosides (27.5%) also contributing substantially. Varying trends in the resistome profiles were observed among other countries, including Canada, Australia, Mexico, Brazil, and Chile. These findings highlight regional differences in resistance gene profiles, underscoring the importance of localized surveillance in understanding and mitigating the spread of antimicrobial resistance globally. Marked geographical variation in ARG abundance was observed across countries; however, these patterns must be interpreted in light of uneven global sequencing coverage. Countries such as the United States, United Kingdom, and China contributed disproportionately large numbers of *Salmonella enterica* genomes to the NCBI Pathogen Detection database, which likely inflates apparent ARG abundance and diversity in these regions. It should be noted that observed regional differences in ARG abundance may partially reflect disparities in the number of *Salmonella enterica* genomes submitted to the NCBI database across geographical regions. North America had the highest abundance of 71.62%, Europe 17.43%, South America 2.89%, Oceania 2.54% and Africa 2.25%.

Conversely, lower ARG counts in parts of Africa, South Asia, and Latin America may reflect limited genomic surveillance rather than true absence or reduced resistance burden. These findings clearly underscore the need for expanded sequencing efforts in underrepresented regions to improve global AMR risk assessment.

### 3.2 Global prevalence of resistance genes demonstrated source-specific resistome signatures

**Fig. 3:**
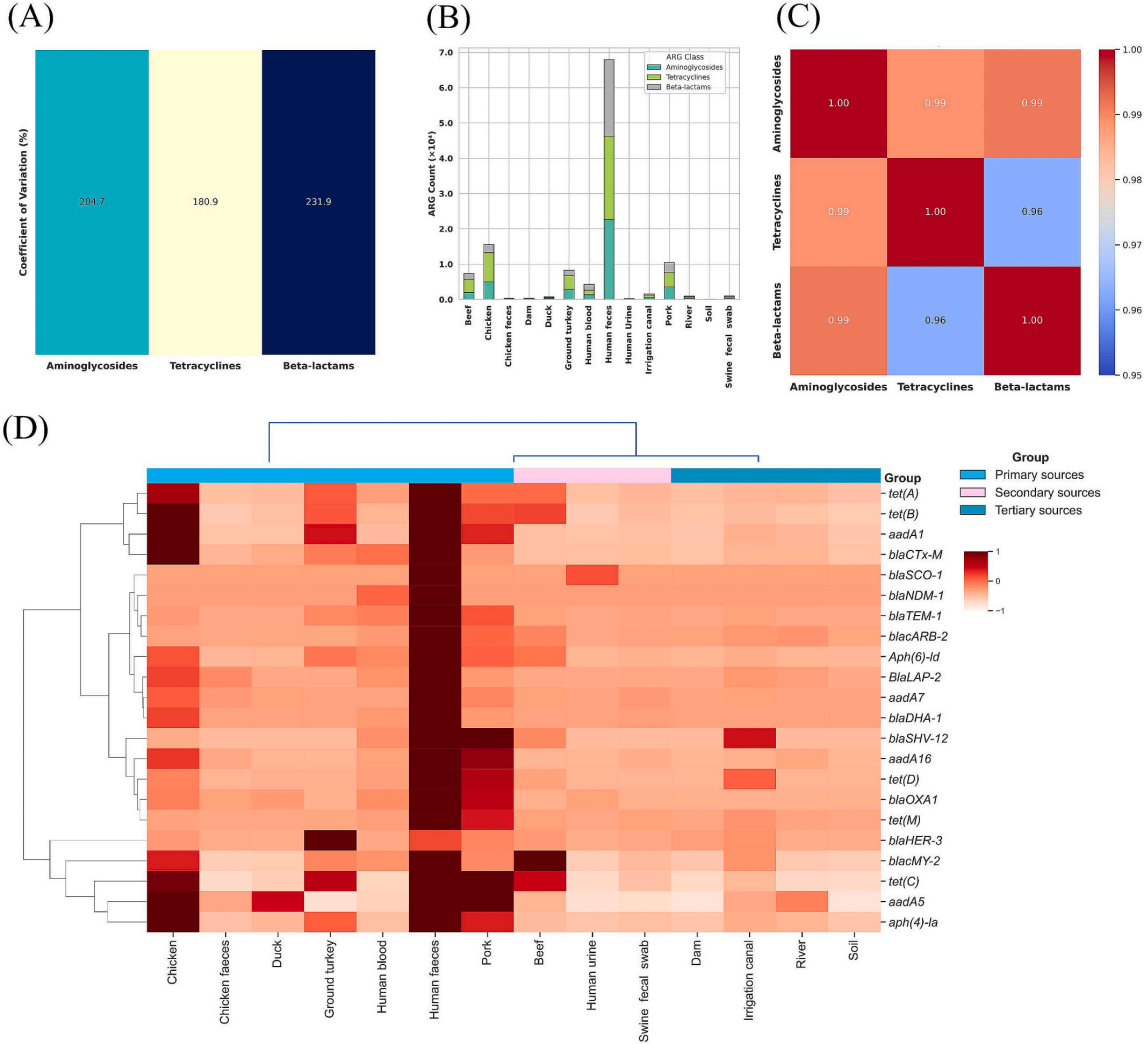
Summary of patterns in ARGs class profiles across diverse isolation sources: (A) The coefficient of variation (CV%) indicates variability in abundance for three major ARG classes, with β-lactams showing the highest variability. (B) ARG class distribution across 14 sample sources, including animal (e.g., chicken, swine), human (e.g., blood, urine), and environmental (e.g., river, soil, dam) origins, with tetracyclines and aminoglycosides were the most abundant (C) Correlation matrix revealing strong positive relationships between ARG classes, especially between aminoglycosides and tetracyclines. (D) Heatmap showing the distribution of antibiotic resistance genes (ARGs) across different sources categorized by transmission risk. The heatmap displays the normalized abundance (*Z*-score) of selected ARGs across various isolation sources, which are grouped into primary, secondary, and tertiary categories based on their potential risk of transmitting ARGs to humans. Hierarchical clustering reveals patterns of ARG occurrence and highlights similarities among different sources.

To gain further insights into ARG profile in respective sources, we performed an integrative multivariate analysis to explore ARG class-level profiles across human, animal, and environmental sources (Fig. 3A–D). Beta-lactam ARGs exhibited the highest variability of 231.9%, followed by aminoglycosides (204.7%) and tetracyclines (180.9%) (Fig. 3A), The high variability, particularly for β-lactam ARGs which were abundant in some sources (e.g., chicken feces) but nearly absent in others (e.g., soil), this may reflect source-specific selection pressures, such as targeted antibiotic use. Human and animal-related sources contributed more to the variation seen in β-lactams. This further suggests that β-lactams, in particular, may be subject to more fluctuating selective pressures across environments. Tetracycline ARGs were relatively more evenly distributed, suggesting they are more widespread or less influenced by individual source environments. We further investigated the distribution of ARG classes in different isolation sources (Fig. 3B). A notable observation of the prevalence of β-lactams in sources like beef and chicken feces, while tetracyclines and aminoglycosides appear more prominent in others like human feces or environmental samples. This raises questions about the specific antibiotic usage in livestock versus human settings and how it influences resistance profiles. We also investigated the similarity and differences between ARG profiles across sources. The results show that sources like human feces and chicken feces cluster closely together, suggesting similar ARG profiles (Fig SB.). In contrast, environmental sources such as river and soil samples are more distantly separated from human and animal samples, indicating that their ARG profiles are quite distinct. This finding points to potential differences in the sources of resistance in various environments, which may be influenced by factors such as local antibiotic use, environmental contamination, and bacterial community composition. ARG class profiles cluster distinctly across different sources, indicating that sources such as beef, chicken, and human feces form separate clusters reflecting distinct ARG profiles. Principal Component Analysis (PCA) of ARG profiles revealed that the first principal component (PC1) explained 98.7% of the variance (Fig.SA). This finding clearly shows that geographic location, animal type, or human contact influences the overall ARG profile in different sources. Lastly, we determined the correlation among ARG classes to understand how strongly different ARG classes (aminoglycosides, tetracyclines, and β-lactams) are related. The high correlation values (0.96) between these ARG classes suggest that resistance mechanisms for one class of antibiotics may be linked to resistance for other classes, a phenomenon called cross-resistance (Fig. 3C). Which could be due to co-resistance, where organisms resistant to one antibiotic also resist others through shared mechanisms such as efflux pumps or enzyme production.

To assess the distribution of antibiotic resistance genes (ARGs) across potential transmission sources, we generated a heatmap of selected ARGs from a range of environmental, animal, and human-associated samples (Fig. 3D). These sources were categorized into primary, secondary, and tertiary groups based on their risk of ARG transmission to humans. Our analysis revealed that primary sources, including human feces, harbored the highest relative abundance and diversity of ARGs. Specifically, human feces showed high counts for *aph(6)-Id* (17,620), *blaTEM-1* (15,479), *tet(A)* (14,645), and *tet(B)* (7246). These findings align with the overall enrichment of β-lactamase genes such as *blaCTX-M*, *blaTEM-1*, and *blaOXA-1*, as well as the widespread presence of tetracycline and aminoglycoside resistance genes like *tet(A)* and *aph(6)-Id* in high-risk sources. Secondary sources, such as pork, chicken, beef and duck, displayed moderate ARG abundance and diversity. Chicken samples contained *aph(6)-Id* (2623), *aph(4)-Ia* (1948), *tet(A)* (4788), and *tet(B)* (3392), while pork samples exhibited *aph(6)-Id* (2354), *blaTEM-1* (1945), *tet(A)* (2004), and *tet(B)* (1496).Tertiary sources including river, soil, and irrigation canals exhibited the lowest ARG abundance and diversity, though sporadic presence of ARGs such as *aph(6)-Id* (329), *aadA1* (157), *blaTEM-1* (202), *tet(A)* (274), and *tet(B)* (212) was observed. Hierarchical clustering analysis of sample resistomes indicated that primary sources clustered together, suggesting shared ARG profiles and potential overlap in exposure routes or microbial communities. Secondary sources formed a separate cluster, while tertiary sources were more heterogeneous. This broad distribution pattern suggests that key resistance mechanisms are not confined to specific environments but have spread across diverse habitats.

Although a subset of ARGs dominated the global *Salmonella enterica* resistome particularly *tet*(A), *aph(6)-Id*, *aadA1,* and *blaTEM-1* less frequently detected variants remain epidemiologically relevant. For example, the detection of *blaTEM-2*, while comparatively rare, reflects historical persistence of early β-lactamase variants within food-associated *Salmonella* lineages. Such genes contribute to the cumulative resistance reservoir and may serve as genetic scaffolds for the evolution or acquisition of more clinically concerning β-lactamases. Therefore, both high- and low-frequency ARGs warrant consideration in surveillance efforts.

Taken together, this study demonstrates that resistome sources critically influence the prevalence of ARGs, which may be due to the influence of some key factors, including microbial community composition, host-associated selection pressures, environmental conditions, the fitness cost of maintaining ARGs, and microbial competition dynamics. Together, these factors drive distinct resistome preferences across different sources. Consequently, these source-specific resistome signatures hold significant potential as biomarkers for tracking the dissemination and evolution of antibiotic resistance.

### 3.3 Food pathway drives resistance gene transmission to humans

**Fig. 4:**
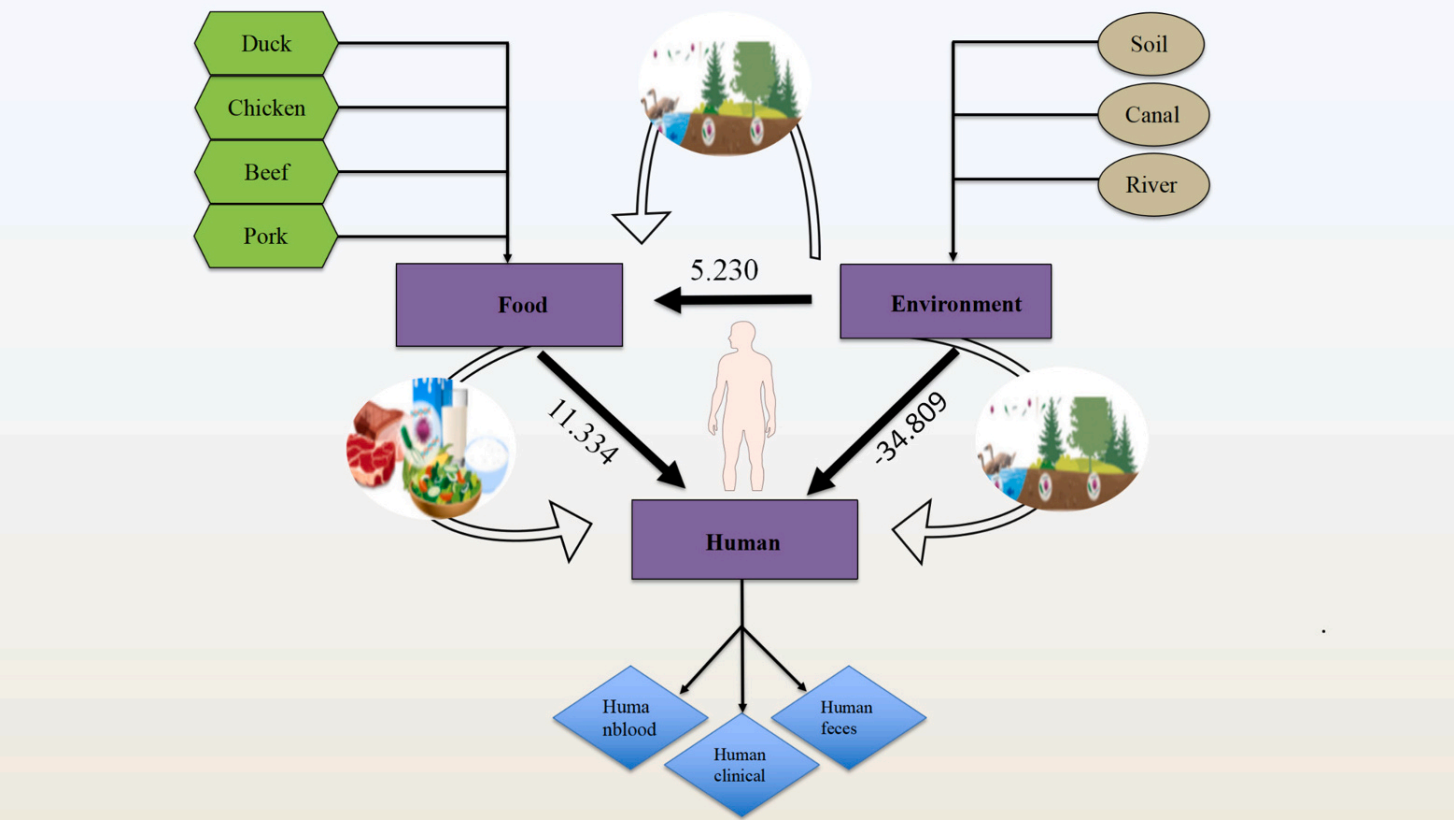
Structural Equation Model (SEM) illustrating the transmission pathways of antibiotic resistance genes (ARGs) from the environment to humans, with food as an intermediate route. ARG data were condensed into three latent categories, Environment, Food, and Humans, by aggregating abundance and diversity metrics across multiple sample types within each domain and standardized resistome profiles.

In this study, we hypothesize that ARGs can be transmitted from food to humans and from human to the environment. The nature of these transmissions was unraveled using the structural equation model (SEM). The structural equation model revealed strong positive associations between environmental and food-associated ARGs and between food-associated and human-associated ARGs within *Salmonella enterica*. In contrast, the direct environment-to-human pathway showed a negative association once food was included as a mediating variable. This pattern suggests that, within the context of *Salmonella enterica*, human exposure to ARGs is more strongly associated with foodborne pathways than with direct environmental contact. Importantly, these results represent statistical associations rather than proof of transmission directionality and should be interpreted accordingly. SEM evaluating the transmission of antibiotic resistance genes (ARGs) from the environment to humans through food revealed a well-fitting model, with CMIN/DF = 0.00 and Comparative Fit Index (CFI) = 1.000, indicating an excellent fit between the hypothesized model and the observed data (Fig. 4). Here we assessed three primary transmission pathways. SEM revealed that the pathway from the environment to food exhibited a strong positive standardized coefficient (β = 5.320, *P* < 0.001), suggesting that ARGs can accumulate in environmental reservoirs such as water, soil, or agricultural surfaces and be subsequently transmitted into the food system. This significant association shows the environment-to-food interface as a critical conduit for resistome movement into the human exposure chain. Furthermore, the food-to-human transmission pathway demonstrated an even stronger positive effect (β = 11.334, *P* < 0.001), indicating a compounding effect whereby ARG concentrations are amplified along the continuum from environmental sources to food products and ultimately to human hosts. This exponential increase in resistome burden across sequential nodes supports the hypothesis that food serves as a primary vehicle for ARG transfer to humans. Notably, the direct environment-to-human pathway exhibited a significant negative standardized coefficient (β = −34.809, *P* < 0.001), implying an inverse relationship when the mediating effect of food is accounted for (Fig. 4). This suggests that, in the presence of food as a mediating variable, the direct influence of environmental ARGs on humans diminishes, potentially due to limited direct exposure or effective barriers at the environment human interface. Collectively, these findings highlight the central role of the food chain in ARG dissemination and emphasize the importance of targeting environmental contamination and food safety as key intervention points for mitigating human exposure to antimicrobial resistance.

## 4 Discussion

Our global analysis of *Salmonella enterica* genomes reveals that antibiotic resistance genes (ARGs) are widely distributed across environmental, food, and human-associated sources, with foodborne pathways playing a central mediating role in resistome connectivity. While ARGs were detected in all compartments, structural equation modeling demonstrated that associations between environmental and human resistomes were strongest when food was included as an intermediate pathway. These findings highlight the importance of food systems in shaping the dissemination of ARGs within *Salmonella enterica* populations associated with human infections, consistent with a One Health framework. These findings offer crucial insights into the complex ecology of resistance, underscoring the urgent need for integrative surveillance and intervention strategies.The analysis of *Salmonella enterica* isolates revealed a widespread distribution of ARGs associated with tetracyclines, aminoglycosides, and β-lactams across diverse geographic regions and isolation sources.Our findings further illustrate distinct geographical differences in the distribution of *S. enterica*, with North America, particularly the United States, leading in isolate counts (Fig. 1). The observed trend reflects a higher surveillance intensity and outbreak documentation, especially within developed nations with robust food production systems [[18], [19], [23]]. The dominance of serotypes such as *S.Enteritidis*, *S.Typhimurium*, and *S.Newport* aligns with global epidemiological studies, and their prevalence in poultry and foodborne outbreaks highlighting the zoonotic potential of these pathogens [[16], [25], [52]]. Regions like South Asia and sub-Saharan Africa showed relatively fewer isolates in our dataset, likely a consequence of limited surveillance, yet these regions bear a higher burden of typhoidal *Salmonella* infections [[1], [42], [43], [57]]. The prevalence of *S. typhi*, *S. paratyphi*, and multidrug-resistant strains like *ST313* of *S. typhimurium* in these regions may result from widespread antibiotic misuse, inadequate sanitation, and limited access to clean water [[2], [27], [32], [48]].

The ARGs analyzed spanned three major antibiotic classes: tetracyclines, aminoglycosides, and β-lactams, with notable genes such as *tet(A), aph(6)-Id*, *aadA1*, and *blaTEM-1* exhibiting high global prevalence.The substantial presence of these genes in both clinical and non-clinical sources highlights the pervasive nature of antibiotic resistance. The global patterns indicate that tetracycline resistance genes were among the most widely disseminated genes,which was consistent with reports from global resistome surveys ([39], [53]). This trend is largely due to the widespread use of tetracyclines in livestock, particularly in food animal production for growth promotion and prophylaxis [[5], [26]]. In contrast, aminoglycoside and beta-lactamase genes vary more regionally, which could be attributed to differences in healthcare infrastructure, regulations, and the extent of antimicrobial usage in both clinical and agricultural sectors. For instance, higher beta-lactamase prevalence in Chile may reflect elevated use of cephalosporins and penicillins in clinical settings [51]. Moreover, countries with stronger regulatory frameworks (e.g., Australia, UK, Canada) tend to show a more balanced ARG distribution and slightly lower beta-lactamase proportions. This supports the role of antimicrobial stewardship and regulatory policy in shaping ARG profiles [55].

Interestingly, while some ARGs were ubiquitous across sources, others showed strong source-specific patterns. For instance, *blaCTX-M,* a critical extended-spectrum beta-lactamase gene, was prevalent in environmental and food sources but also found extensively in clinical isolates, a pattern suggesting its role in bridging environmental and human reservoirs [50]. Similarly, *aph(4)* and *aadA2* were enriched in animal and environmental samples, supporting the hypothesis that agricultural and animal husbandry practices contribute significantly to ARG propagation [34].

Moreover, the detection of carbapenemase-producing *S. enterica* strains in clinical settings, notably *blaNDM-1*, raises alarm, as these ARGs are often acquired through environmental vectors and are resistant to last-resort antibiotics [40]. The uneven distribution of ARGs across different sources reinforces the concept of a tiered risk system in the transmission of antimicrobial resistance to humans. The high ARG burden in primary sources, particularly in human feces and chicken feces, highlights their role as major reservoirs and potential contributors to the clinical resistome. These findings are in line with previous studies showing elevated ARG levels in the gut microbiota of humans and food-producing animals [21].

The co-occurrence of multiple ARGs within single isolates, particularly in food and clinical strains, points to horizontal gene transfer (HGT) as a critical mechanism in resistance spread. Mobile genetic elements such as plasmids, integrons, and transposons have been implicated in the simultaneous acquisition and dissemination of multiple resistance traits [11], [44], [49]. The plasmid-mediated transmission of genes like *tet(M)* and *blaTEM-1* likely facilitates their movement across bacterial species and environmental niches, compounding the challenge of resistance management. The detection of 22 shared ARGs, including *tet(A), blaCTX-M*, and *blaTEM-1*, across environmental and human samples suggests an active transmission cycle, possibly facilitated by environmental contamination [3].

The developed structural equation model (SEM) provided clear insights into the pathways of ARG transmission and aligns with the One Health framework, which recognizes the interconnectedness of human, animal, and environmental health in antimicrobial resistance (AMR) dynamics [[47], [65]]. The significant environment-to-food relationship revealed that when the environmental ARGs increase by 1 factor, food increases by standardized coefficient of 5.230, which highlights the role of environmental reservoirs such as wastewater and contaminated soil in introducing ARGs into the food chain. Aplethora of studies have also shown that ARGs present in irrigation water, manure-fertilized fields, and surface runoff can be taken up by crops or transferred to food animals [3], [12].The environmental contamination serves as a primary route by which resistance enters the human food chain.The strong, food-to-human pathway (11.134) suggested that foodborne exposure is the principal pathway by which ARGs reach human populations.This finding is supported by multiple studies identifying resistant bacteria and genes in raw meats, vegetables, and ready-to-eat products [31], [46].Moreover, the use of antibiotics in livestock and aquaculture further increases the risk of ARGs entering the food supply [34]. This emphasizes the need for strengthened food safety regulations and monitoring systems targeting both pre and post-harvest contamination routes [7].

The negative coefficient from environment to human pathway (−34.809) may suggest that while the environment is an origin of ARGs, its impact on humans is overwhelmingly indirect, operating primarily through foodborne pathways.Alternatively, this may point to an attenuated direct transmission pathway due to limited human contact with environmental reservoirs in some settings, or strong food-centric transmission overshadowing other pathways.This interpretation aligns with recent studies emphasizing the importance of food and animal contact in ARG transfer compared to environmental exposure alone [[13], [30]].

Our findings further sheds light on the diversity and variability of ARG profiles across different environmental and host-associated isolation sources. The high coefficient of variation (CV) for beta-lactams (231.9%) underscores their inconsistent presence in the environment, likely driven by differential antibiotic usage and selective pressures [11](Fig. 3A). The variability observed in aminoglycosides and tetracyclines is similarly notable, suggesting that the persistence of these ARGs may be influenced by localized factors such as agricultural practices or water contamination [64]. The distribution of ARG classes (Fig. 3B), shows a clear dominance of tetracyclines and beta-lactams in human-associated environments, particularly human feces, aligns with findings from previous studies that have highlighted these environments as significant reservoirs for ARGs [19]. In contrast, animal sources, such as beef and chicken, exhibit lower total counts of ARGs, which may reflect reduced antibiotic pressure or more localized resistance patterns compared to human feces.

The distinct separation of samples in NMDS ordination (Fig.SB) and PCA (Fig.SA) further supports the idea of source-specific resistomes, with swine fecal swabs and human urine exhibiting unique ARG profiles compared to other sources. This is consistent with previous studies indicating that swine, as well as poultry, are reservoirs of multidrug-resistant bacteria due to intensive antibiotic use in agricultural settings [60].

The strong correlations observed between aminoglycosides, tetracyclines, and beta-lactams (Fig. 3C) suggest significant co-selection of resistance mechanisms, which may be facilitated by the spread of multidrug-resistant plasmids or other mobile genetic elements across both human and environmental reservoirs [15], [44]. These correlations clearly highlight the importance of monitoring multiple classes of resistance genes simultaneously, as they may provide a more comprehensive understanding of ARG transmission dynamics.

The findings of the present study further signal the urgent need for integrated, source-attributed surveillance systems that encompass human, animal, and environmental health sectors, a core principle of the One Health approach. Improved monitoring in underrepresented regions such as parts of Africa, Asia, and Latin America is vital to accurately assess and respond to ARG dissemination.Moreover, the identification of specific ARGs as high-risk (e.g *blaCTX-M*, *blaNDM-1*) can inform targeted intervention strategies and stewardship programs aimed at curbing antibiotic misuse in agriculture and healthcare.

Although our study leveraged a robust global dataset, the uneven distribution of sequencing data across regions and isolation sources may introduce bias in ARG prevalence estimates. Additionally, while statistical associations suggest possible transmission routes, causality and specific mechanisms require experimental validation. Future studies should incorporate metagenomic analyses, longitudinal sampling, and plasmid tracking to better characterize ARG mobility and transmission dynamics across ecosystems.

## 5 Conclusion

The present study provides global-scale insights into the distribution and transmission of antibiotic resistance genes within *Salmonella enterica*. We demonstrate that clinically relevant ARGs conferring resistance to aminoglycosides, tetracyclines, and β-lactams are widespread across environmental, food, and human-associated sources. Distinct source-specific resistome signatures were observed, with primary sources exhibiting the highest ARG abundance and diversity, while food sources functioned as key intermediates linking environmental reservoirs to human-associated *Salmonella*.

Structural equation modeling indicates that foodborne pathways play an important mediating role in the dissemination of ARGs within *Salmonella enterica* associated with human infections, whereas direct environmental exposure appears comparatively limited when food is considered. These findings emphasize the relevance of food systems in *Salmonella*-associated AMR dynamics and support integrated One Health surveillance strategies targeting food production, agricultural practices, and environmental contamination. Future studies incorporating longitudinal sampling, metagenomics, and mobile genetic element tracking will be essential to further elucidate transmission mechanisms and clinical impact. Expanding surveillance in underrepresented regions will also be vital for a more accurate global understanding of resistance dynamics and for guiding policy and mitigation strategies.

## CRediT authorship contribution statement

**Emmanuel Stephen Odinga:** Writing – review & editing, Writing – original draft, Visualization, Formal analysis, Conceptualization. **Okugbe Ebiotubo Ohore:** Writing – review & editing, Writing – original draft, Visualization, Validation, Supervision, Funding acquisition, Formal analysis, Data curation, Conceptualization. **Sanji Zhou:** Software, Data curation. **Zhao Ziyu:** Software, Data curation. **Zhang Hongrui:** Software. **Guojing Yang:** Writing – review & editing, Writing – original draft, Supervision, Funding acquisition, Conceptualization.

## Declaration of competing interest

The authors declare that they have no known competing financial interests or personal relationships that could have appeared to influence the work reported in this paper.

## Data Availability

Data will be made available on request.
