# Supplementary material for: Global transmission dynamics of antibiotic resistance genes: Foodborne pathways as the critical link to humans
Source: One Health. 2026 Jul 25;23:101531. doi: 10.1016/j.onehlt.2026.101531 (PMC13452398; doi:10.1016/j.onehlt.2026.101531)
Supplement: Supplementary file 1 [file mmc1.docx]

**Supplementary Information**

**Global transmission dynamics of antibiotic resistance genes: foodborne pathways as the critical link to humans**

Emmanuel Stephen Odinga^1^ Okugbe Ebiotubo Ohore^1^, Sanji Zhou, Zhao Ziyu, Zhang Hongrui, Guojing Yang^*^

Key Laboratory of Tropical Translational Medicine of Ministry of Education, School of Public Health, Hainan Academy of Medical Sciences, Hainan Medical University, Haikou, Hainan, 571199, China.

***Corresponding Author:**

**Guo-Jing Yang** (yangguojing@hainmc.edu.cn)

1 These authors contributed equally towards this paper.

**Figures Captions**

**Figure S1. Distribution patterns of antibiotic resistance genes (ARGs) globally.** Geographic distribution of samples with ARG abundance in various habitats. Each point indicates one sampling location, with point size , and point color indicating the ARGs count.

**Figure S2.** (A) Principal Component Analysis (PCA) of ARG class profiles, with most variation explained by the first principal component (PC1).

(B) Non-metric multidimensional scaling (NMDS) ordination based on Bray-Curtis dissimilarity highlights source-specific clustering, suggesting differences in ARG class composition among sample types.

**Figure S1.**

**Figure S2.**

**Table Captions**

**Table S1. Distribution of aminoglycosides, resistance genes across various sample sources classified by transmission risk level**. The table present the total counts of respective ARGs classes detected in samples from different origins including human, animal, and environmental sources.

**Table S2. Distribution of tetracyclines, resistance genes across various sample sources classified by transmission risk level**. The table present the total counts of respective ARGs classes detected in samples from different origins including human, animal, and environmental sources.

**Table S3.** **Distribution of beta lactams resistance genes across various sample sources classified by transmission risk level.** The table present the respective ARGs classes detected in samples from different origins including human, animal, and environmental sources.

**Table S1.**

| **Source** | **Classification** | ***Aph(6)-ld*** | ***aadA1*** | ***aadA16*** | ***aadA5*** | ***aadA7*** | **Total** |
| --- | --- | --- | --- | --- | --- | --- | --- |
| Beef | Secondary | 1905 | 61 | 1 | 11 | 4 | 1982 |
| Chicken | Primary | 2623 | 2121 | 42 | 72 | 107 | 4965 |
| Chicken feces | Primary | 42 | 16 | 5 | 14 | 18 | 95 |
| Dam | Tertiary | 72 | 13 | 0 | 0 | 1 | 86 |
| Duck | Secondary | 121 | 31 | 1 | 39 | 5 | 197 |
| Ground turkey | Secondary | 1815 | 1080 | 1 | 1 | 3 | 2900 |
| Human blood | Primary | 1359 | 87 | 8 | 5 | 3 | 1462 |
| Human feces | Primary | 17620 | 3795 | 223 | 93 | 971 | 22702 |
| Human urine | Secondary | 62 | 20 | 0 | 1 | 0 | 83 |
| Irrigation canal | Tertiary | 329 | 157 | 2 | 14 | 4 | 506 |
| Pork | Secondary | 2354 | 995 | 74 | 72 | 47 | 3542 |
| River | Tertiary | 148 | 115 | 6 | 21 | 0 | 290 |
| Soil | Tertiary | 7 | 2 | 0 | 0 | 0 | 9 |
| Swine fecal swab | Secondary | 232 | 51 | 3 | 2 | 15 | 303 |

**Table S2.**

| **Source** | **Classification** | ***tet(A)*** | ***tet(B)*** | ***tet(C)*** | ***tet(D)*** | ***tet(M)*** | **Total** |
| --- | --- | --- | --- | --- | --- | --- | --- |
| Beef | Secondary | 2069 | 1550 | 115 | 5 | 15 | 3754 |
| Chicken | Secondary | 4788 | 3392 | 156 | 14 | 11 | 8361 |
| Chicken feces | Primary | 48 | 17 | 0 | 0 | 2 | 67 |
| Dam | Tertiary | 76 | 76 | 3 | 0 | 1 | 156 |
| Duck | Primary | 136 | 145 | 10 | 1 | 3 | 295 |
| Ground turkey | Secondary | 2413 | 1382 | 122 | 1 | 5 | 3923 |
| Human blood | Primary | 849 | 324 | 5 | 6 | 19 | 1203 |
| Human feces | Primary | 14645 | 7246 | 185 | 177 | 1317 | 23570 |
| Human urine | Secondary | 31 | 21 | 0 | 0 | 2 | 54 |
| Irrigation canal | Tertiary | 274 | 212 | 24 | 23 | 40 | 573 |
| Pork | Secondary | 2004 | 1496 | 313 | 49 | 255 | 4117 |
| River | Tertiary | 346 | 103 | 4 | 0 | 8 | 461 |
| Soil | Tertiary | 4 | 1 | 1 | 0 | 1 | 7 |
| Swine fecal swab | Secondary | 235 | 200 | 18 | 0 | 12 | 465 |

**Table S3.**

| **Source** | **Classification** | ***blaCTx-M*** | ***blaDHA-1*** | ***blaHER-3*** | ***blaNDM-1*** | ***blaOXA1*** | ***blaSCO-1*** | ***blaSHV-12*** | ***blaTEM-1*** | ***blaCARB-2*** | ***blacMY-2*** | ***blaLAP-2*** |
| --- | --- | --- | --- | --- | --- | --- | --- | --- | --- | --- | --- | --- |
| Beef | Secondary | 21 | 0 | 4 | 0 | 4 | 0 | 9 | 205 | 91 | 1222 | 0 |
| Chicken | Secondary | 1344 | 15 | 4 | 0 | 111 | 0 | 3 | 372 | 12 | 337 | 16 |
| Chicken feces | Secondary | 54 | 0 | 0 | 0 | 35 | 0 | 0 | 49 | 0 | 0 | 5 |
| Dam | Tertiary | 2 | 0 | 3 | 0 | 2 | 0 | 0 | 44 | 14 | 16 | 0 |
| Duck | Secondary | 93 | 0 | 0 | 0 | 49 | 0 | 0 | 80 | 3 | 7 | 0 |
| Ground turkey | Secondary | 264 | 0 | 150 | 0 | 0 | 0 | 0 | 845 | 5 | 161 | 0 |
| Human blood | Primary | 307 | 2 | 1 | 2 | 78 | 0 | 8 | 1046 | 35 | 133 | 4 |
| Human feces | Primary | 1944 | 107 | 22 | 22 | 1359 | 90 | 99 | 15479 | 1724 | 666 | 109 |
| Human urine | Secondary | 19 | 0 | 0 | 0 | 33 | 11 | 0 | 42 | 1 | 1 | 0 |
| Irrigation canal | Tertiary | 61 | 0 | 6 | 0 | 3 | 0 | 25 | 202 | 41 | 128 | 3 |
| Pork | Secondary | 159 | 2 | 9 | 0 | 353 | 0 | 41 | 1945 | 173 | 150 | 3 |
| River | Tertiary | 55 | 0 | 0 | 0 | 0 | 0 | 0 | 85 | 47 | 12 | 2 |
| Soil | Tertiary | 1 | 0 | 0 | 0 | 2 | 0 | 0 | 5 | 2 | 1 | 0 |
| Swine fecal swab | Secondary | 23 | 1 | 1 | 0 | 3 | 0 | 0 | 133 | 12 | 47 | 0 |
